# The Influences of Tai Chi on Balance Function and Exercise Capacity among Stroke Patients: A Meta-Analysis

**DOI:** 10.1155/2021/6636847

**Published:** 2021-02-25

**Authors:** Xinhu Zheng, Xiaoyang Wu, Zuhong Liu, Jing Wang, Keyang Wang, Jilin Yin, Xing Wang

**Affiliations:** ^1^Shanghai University of Sport, Shanghai 200438, China; ^2^Jiaxing University, Jiaxing 314001, China; ^3^Shanghai Lixin University of Accounting and Finance, Shanghai 201620, China; ^4^Shanghai University of Political Science and Law, Shanghai 201207, China; ^5^Bei Gulf University, Qinzhou 535011, China

## Abstract

**Objective:**

This study aims to explore the influences of Tai Chi on the balance function and exercise capacity among stroke patients.

**Methods:**

Databases including PubMed, Embase, WOS (Web of Science), the Cochrane Library, CNKI (China National Knowledge Infrastructure), Wanfang Data, VIP (VIP database), and CBM (China Biology Medicine disc) were retrieved to gather the figures of randomized controlled trials on the balance function and exercise capacity among stroke patients. Then relevant data were input and analyzed in Review Manager 5.3.

**Results:**

Nineteen papers were included and analyzed in this study. According to the combined effect size, the balance function of stroke patients improved significantly: the Berg Balance Function Scale score [MD = 7.67, 95% CI (3.44, 11.90)]; standing and walking test scores [MD = 3.42, 95% CI (4.22, −2.63)]; gravity swing area [MD = 0.79, 95% CI (1.48, 0.10)]; and gravity swing speed [MD = −5.43, 95% CI (−7.79, 3.08)]. In addition, the exercise capacity improved significantly as well: the FMA (Fugl-Meyer Assessment Scale) scale score [MD = 4.15, 95% CI (1.68, 6.63)]. There are no significant influences or changes of other related results.

**Conclusions:**

Stroke patients are able to improve their balance functions and exercise capacities prominently when they do Tai Chi exercise once or twice a week and ≥5 times/week and >30 ≤ 60 min/time.

## 1. Introduction

Stroke, a common and frequently occurring disease among middle-aged and elderly people, is a disease of brain tissue damage caused by the sudden rupture of cerebral blood vessels or vascular obstruction, which has high incidence, high disability rate, and high mortality rate [1]. Hemiplegia is one of the most common sequelae of stroke, whose patients usually lose muscle strength and balance function of one limb, making them inconvenient or unable to move at all [2]. According to statistics, there are about two million new stroke patients every year in China; nowadays, there are a total of 6–7 million survival Chinese patients, whose mortality rate reaches 10%–30% and the disability rate is around 60%–70%. In addition, around 80% of stroke patients suffer from impairment of lower limb motor function [3], which severely troubles their daily activities and lowers their quality of life [4].

As a low-intensity aerobic exercise, Tai Chi is a safe, effective, and inexpensive adjuvant therapy and rehabilitation method [5] and has been reported many times in recent years for being applied in daily rehabilitation of patients with chronic diseases and the elderly [6]. The evidence of quantitative research shows that Tai Chi can relax the tense muscles of patients, enhance their flexibility and strength, inhibit the occurrence of abnormal postures and spasms, improve the balance function, enhance the normal exercise ability and control ability of stroke patients, and have many positive effects on the daily activities and psychological emotions of stroke patients [7, 8], thus improving the life quality of stroke patients.

In previous studies, none of the randomized controlled trial studies on Tai Chi intervention have been integrated to analyze the role of Tai Chi on stroke. Therefore, this study aims to clarify the effect of Tai Chi on the balance function and exercise ability among stroke patients, as well as illustrate the influences of various exercise variables among the patients, so as to provide references for the development of precise exercise programs.

## 2. Research Method

This study followed the requirements of the international meta-analysis writing guidelines (the PRISMA statement for reporting systematic reviews and meta-analyses of studies that evaluate health-care interventions: explanation and elaboration) [9] for the selection and use of research methods. The protocol for this study was registered with INPLASY (202110086).

### 2.1. Literature Inclusion and Exclusion Criteria

#### 2.1.1. Research Design

A meta-analysis of randomized controlled trials (RCT) and the influences of Tai Chi on balance function and exercise capacity among stroke patients is conducted.

#### 2.1.2. Criteria of the Included Literature

① The subjects of the study were all stroke patients with stable conditions and were in line with the stroke diagnostic criteria formulated by the 4th Cerebrovascular Disease Academic Conference of the Chinese Medical Association [10] or the stroke diagnostic criteria regulated by the American Heart Association/American Stroke Association (AHA/ASA) [11]; ② the vital signs were stable; ③ if a study had two treatment groups, it was regarded as two studies; and ④ the type of this study is randomized controlled trial (RCT).

#### 2.1.3. Criteria of the Excluded Literature

① The subjects included in the experiment were normal elderly without stroke diseases; ② the subjects had dyskinesias and could not complete Tai Chi exercises; ③ there was no pure Tai Chi exercise intervention group; ④ papers with multiple releases and low-quality assessment; and ⑤ papers with unclear and incalculable experimental data.

### 2.2. Literature Retrieval Strategy

Databases including PubMed, the Cochrane Library, Embase, Web of Science (WOS), China National Knowledge Infrastructure (CNKI), Wanfang Data, VIP, and China Biology Medicine disc (CBM) were retrieved to gather randomized controlled trial figures on balance function and exercise mobility on stroke patients who did Tai Chi exercise. The retrieval period started from the initial period of each database to June 30, 2020. The search strategy was based on the principle of PICOS (Population, Intervention, Comparison, Outcomes, and Study design) and adopted a combination of subject words and free words, which were determined after repeated prechecks, supplemented by manual search and the references tracking of those papers when necessary. Chinese search terms include cerebral stroke (脑卒中), stroke (中风), cerebral thrombosis (脑血栓), cerebral infarction (脑梗死), Tai Chi (太极), Tai Chi Chuan (太极拳), random (随机), and experiment (试验). The English search terms used the Web of Science database as an example:

  #1 TS = (stroke or apoplexy or cerebrovascular accident)
  #2 TS = (Tai Ji or Tai-ji or Tai Chi or Tai Ji Quan or Taiji or Taijiquan or Tai Chi Chuan)
  #3 TS = (balance or equilibrium or posture control or posture reaction or athletic ability or exercise performance or motor ability or exercise capacity or sports ability)
  #4 TS = (randomized controlled trial or randomized or controlled or trial)
   #5 #1 and #2 and #3 and #4

#### 2.2.1. Intervention Measures

At least, one group only used Tai Chi exercise as the intervention method, and the control group received placebo, health education, daily activities, routine nursing, etc.

#### 2.2.2. Outcome Indicators

Outcome indicators selected various scales and testing indicators for evaluating balance function and exercise ability, including the Berg Balance Scale (BBS), standing and walking, gravity center swing, short physical performance battery (SPPB), Fugl-Meyer Assessment Scale (FMA), and six-minute walking test (6MWT).

### 2.3. Literature Screening, Data Extraction, and Quality Evaluation

#### 2.3.1. Literature Screening

Two researchers used independent double-blind methods to screen the literature based on the criteria of the included and excluded literature and extracted relevant data. If there was a disagreement on the mutual review, screening, and data extraction phases, a third researcher would join in to discuss whether to include the data.

#### 2.3.2. Data Extraction

The data extracted from the literature mainly include the author's name, year of publication, nationality, sample size, age, exercise form, exercise cycle, exercise duration, exercise frequency, and exercise intensity. The outcome indicator data extracted from the study were the change values of outcome index included in the literature, that is, the postintervention test data subtracted the preintervention test data.

Physical exercise variables were divided based on relevant previous researches: exercise duration lasting for ≤30 min (minutes), >30 ≤ 60 min, and >60 min [12]; exercise intensity had low, medium, and high categories [13]; exercise frequency included 1‐2 times/wk (times per week), 3‐4 times/wk, and ≥5 times/wk, and the cycle was divided into ≤12 weeks, >12 ≤ 24 weeks, and >24 weeks [14].

#### 2.3.3. Quality Evaluation

The risk of bias criteria of randomized controlled trials (RCT) in the Cochrane Collaborative Network were adopted to perform qualitative evaluation of seven aspects of RCT: random sequence generation, distribution concealment, blind method of subjects and researchers, blind method of outcome evaluator, incomplete outcome data, selective report, and other bias, and each index was judged by “low bias risk,” “uncertain bias risk,” or “high bias risk.”

#### 2.3.4. Level of Evidence

In this study, the evidence level of the included literature was graded according to the evidence level of the Oxford Center for Evidence-Based Medicine [15] (Table 1).

#### 2.3.5. Statistical Analysis

Using Review Manager 5.3 for the literature data process, this paper had the combined effect size and heterogeneity test and drew a forest diagram. The literature outcome indicators were all continuous variables, the effect size chose Mean Difference (MD) and Standardized Mean Difference (SMD), and the effect size was MD = 95% of confidence interval. This meta-analysis strictly follows the PRISMA guidelines [8] and used the *P* value and I2 for the heterogeneity test. If there was no statistical heterogeneity between the results of each study (I2 ≤ 50%, *P* > 0.10), the fixed-effects model would be selected. If there was statistical heterogeneity between the studies, the source of the heterogeneity would be further analyzed, and the random effects model was used for analyses after excluding the influence of obvious clinical heterogeneity.

## 3. Results

### 3.1. Process and Results of Literature Screening

**Figure 1:**
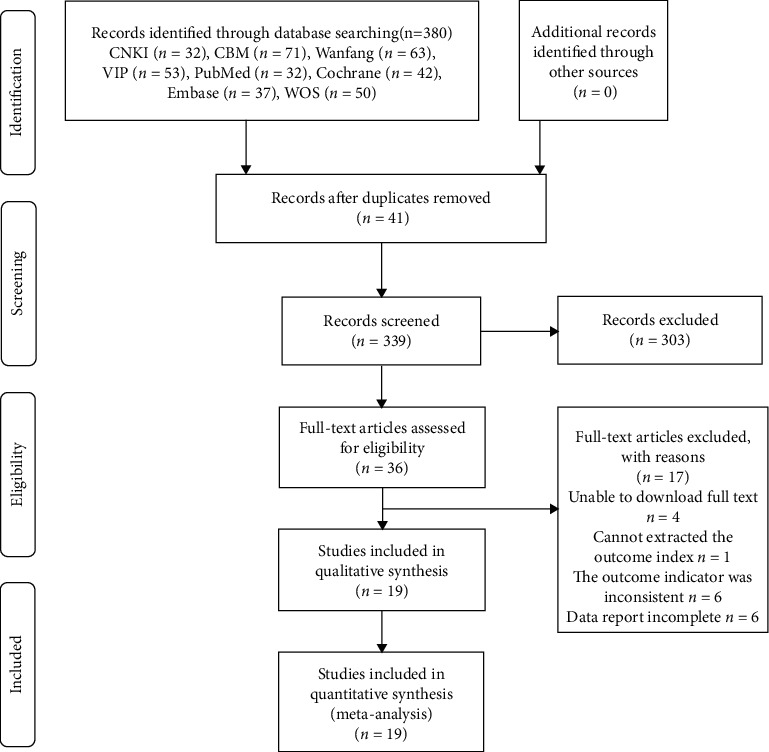
Literature screening process.

Initially, a total of 380 articles were retrieved from the database, among which 41 repeated papers were excluded. According to the inclusion and exclusion criteria, 303 papers were screened and excluded. Then, 17 articles were excluded after a full-text screening. At last, 19 articles were left (Figure 1).

### 3.2. The Included Literature

#### 3.2.1. Basic Information of the Included Literature

Nineteen randomized controlled trial (RCT) literature were included, whose subjects were all stroke patients. According to the evidence level standard and recommendation level of the Oxford Center for Evidence-Based Medicine, the evidence levels of the included literature were all Ib, which stands for a relatively high evidence level and good quality (Table 2).

#### 3.2.2. Sports Intervention Features in the Included Literature

Tai Chi, as an intervention method, was adopted in all the 19 included papers. Their selected intervention period lasted 12 weeks or less, and there were two articles that did not report the intervention period. Four papers chose the intervention frequency as twice a week, six papers chose 3‐4 times/wk, and nine papers chose ≥5 times/wk. Four papers had the exercise duration ≤30 min per time, 14 papers had 30–60 min per time, and one paper had >60 min per time. The research results in the included literature were all the balance function and exercise ability among stroke patients (Table 3).

#### 3.2.3. Bias Risk Analysis of the Included Literature

**Figure 2:**
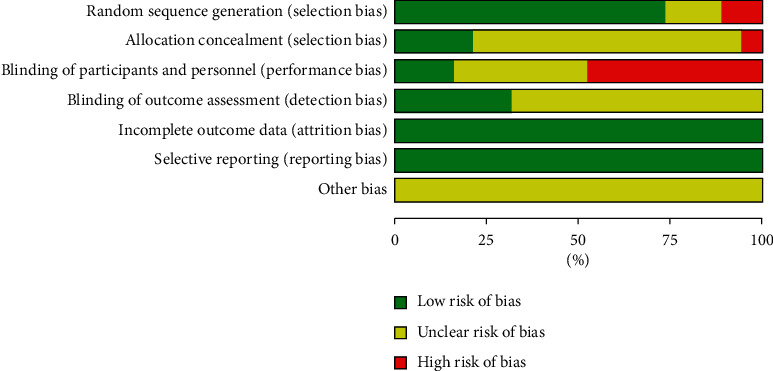
The overall risk of bias of the included papers.

**Figure 3:**
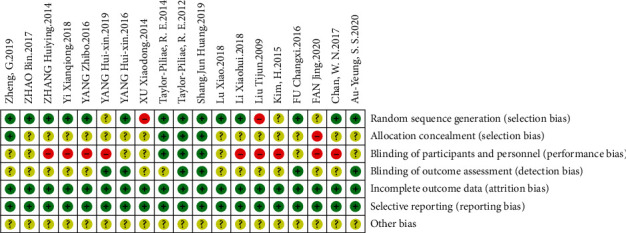
The risk of bias of the included papers.

As shown in Figures 2 and 3, Review Manager software was used to analyze the risk of bias in the included literature. Among these papers, 5 papers did not explain the method of random allocation of subjects, and only 4 papers used the method of allocation and hiding to randomly divide the subjects and did not blind the subjects. Only 3 papers blinded the subjects and 6 papers conducted the blinding of evaluation, among which 2 papers conducted double-blinded experiments on both subjects and result evaluation. Data of the 19 papers were complete, and other sources of bias risk were not explained.

### 3.3. Meta-Analysis Results

#### 3.3.1. The Influences of Tai Chi on the Berg Balance Scale

**Figure 4:**
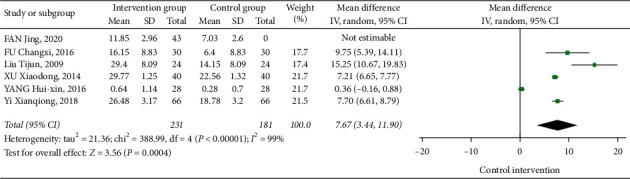
The forest diagram of the influences of Tai Chi on the BBS.

Six papers studied the influence of Tai Chi on Berg Scale scores. The heterogeneity analysis showed that *I*^2^ = 99%, *P* < 0.01, showing heterogeneity features in those studies. Therefore, the random effects model was used to analyze the results, and the combined effect size showed [MD = 7.67, 95% CI (3.44, 11.90)]. Compared with the control group, Tai Chi can significantly improve the Berg Balance Scale score of stroke patients (Figure 4).

#### 3.3.2. The Influences of Tai Chi on the Standing and Walking Test

**Figure 5:**
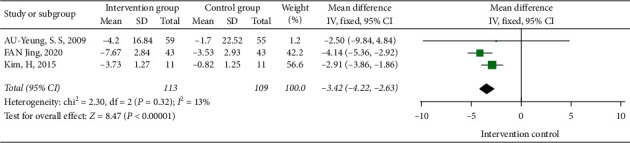
Forest diagram of the influences of Tai Chi on the standing and walking test.

Three papers studied the influence of Tai Chi on the standing and walking test of stroke patients. The analysis of heterogeneity showed that *I*^2^ = 13%, *P*=0.32 > 0.05. In other words, there was no statistical heterogeneity between those studies. Thus, the fixed-effects model was selected to analyze, and the meta-analysis results were [MD = −3.42, 95% CI (−4.22, −2.63)], indicating that Tai Chi can significantly improve the standing and walking ability of stroke patients (Figure 5).

#### 3.3.3. The Influence of the 6 m Walking Test on the Patients Doing Tai Chi

**Figure 6:**
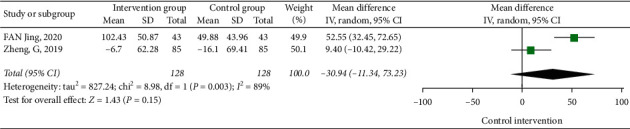
Forest diagram of the influences of Tai Chi on the 6 m walking experiment.

Two papers studied the influences of Tai Chi on the 6 m walking experiment. The heterogeneity results showed that *I*^2^ = 89%, *P*=0.003 < 0.05, so the random effects model was selected to conduct meta-analysis of the results, and the results showed that the combined effect size was [MD = 30.94, 95% CI (−11.34, 73.23)]. Tai Chi can significantly improve the 6 m walking distance of stroke patients, but the difference is not significant (Figure 6).

#### 3.3.4. The Influence of Tai Chi on the Swing of the Gravity Center

**Figure 7:**
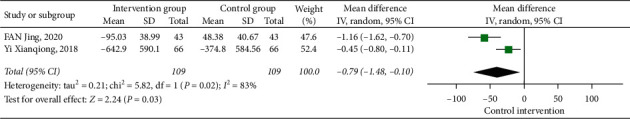
Forest diagram of the influences of Tai Chi on the swing area of the gravity center.

**Figure 8:**
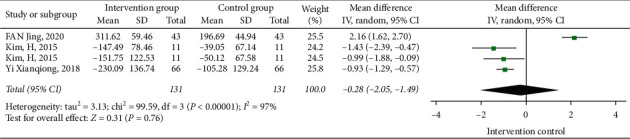
Forest diagram of the influences of Tai Chi on the swing length of the gravity center.

**Figure 9:**
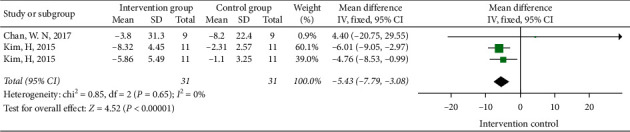
The forest diagram of the influences of Tai Chi on the swing speed of the gravity center.

Two papers studied the influence of Tai Chi on the swing area of the center of gravity (Figure 7), three papers studied the effect of Tai Chi on the swing length of the center of gravity (Figure 8), and two papers studied the effect of Tai Chi on the swing speed of the center of gravity (Figure 9). The heterogeneity test results were *I*^2^ = 83%, *P*=0.02 < 0.05; *I*^2^ = 97%, *P*=<0.01; and *I*^2^ = 0%, *P*=0.65 > 0.05. Therefore, the random effect model was selected in the tests of swing area of gravity center and swing length of gravity center, and the fixed-effects model was selected in the swing speed of the gravity center test to conduct meta-analysis on the results. The swing area of gravity center was [MD = −0.79, 95% CI (−1.48, −0.10)], swing length of gravity center was [MD = −0.28, 95% CI (−2.05, 1.49)], and swing speed of gravity center was [MD = −5.43, 95% CI (−7.79, −3.08)]. The results show that Tai Chi can significantly improve the area and speed of the gravity center swing of stroke patients while standing, but it has no statistical significance on the length of the gravity center swing.

#### 3.3.5. The Influences of Tai Chi on FMA

Ten papers analyzed the influence of Tai Chi on the FMA scale. The heterogeneity analysis results showed that *I*^2^ = 98%, *P* < 0.01. Therefore, a meta-analysis of the results using a random effects model showed that [MD = 4.15, 95% CI (1.68, 6.63)]. Compared with the control group, Tai Chi can significantly improve the FMA scale scores of stroke patients, or the exercise ability improved significantly.

**Figure 10:**
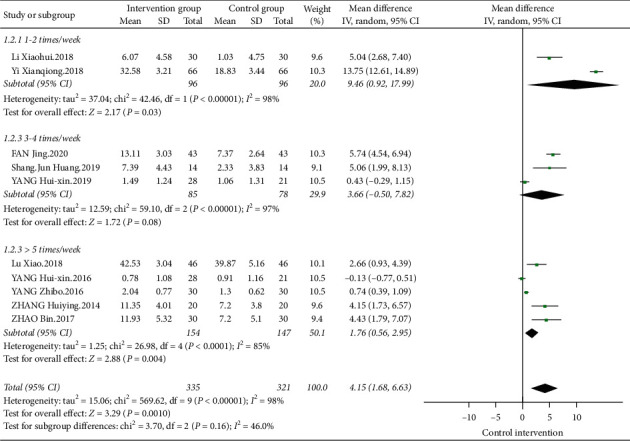
Forest diagram of the influences of different exercise frequencies on FMA.

Two papers selected once-twice/wk exercise frequency for Tai Chi intervention, whose combined effect size showed [MD = 9.46, 95% CI (0.92, 17.99)]. Three studies selected 3–4 times/wk exercise frequency for Tai Chi exercise, whose combined effect size showed [MD = 3.66, 95% CI (−0.50, 7.82)]. Five papers studied exercise frequency ≥5 times/wk, whose combined effect size was [MD = 1.76, 95% CI (0.56, 2.95)]. The results show that 3–4 times/wk of Tai Chi exercises cannot significantly improve the FMA scale scores of patients, while once-twice/wk and ≥5 times/wk of Tai Chi exercises can significantly improve FMA scores and improve exercise ability of patients (Figure 10).

**Figure 11:**
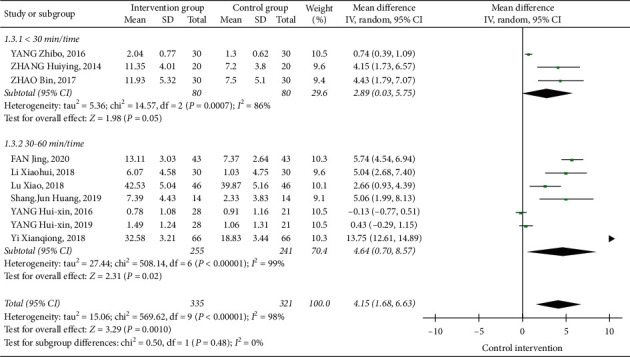
Forest diagram of the influences of different exercise durations on FMA.

Three papers selected Tai Chi exercises lasting for ≤30 min/time, whose combined effect size was [MD = 2.89, 95% CI (0.03, 5.75)]. Seven papers studied 30–60 min per time Taijiquan exercises, whose combined the effect size was [MD = 4.64, 95% CI (0.70, 8.57)]. The results show that both kinds of exercise duration can improve the FMA scale score and improve exercise ability, while the improvement effect of 30–60 min/time exercise time is better (Figure 11).

#### 3.3.6. The Impact of Tai Chi on SPPB

**Figure 12:**
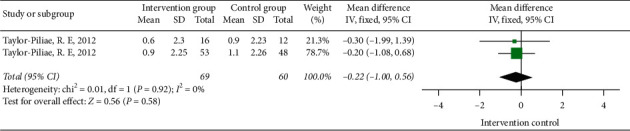
The forest diagram of the impact of Tai Chi on SPPB.

Two papers studied the influence of Tai Chi on the SPPB scale. The heterogeneity analysis showed that *I*^2^ = 0%, *P*=0.92 > 0.05, that is, the two studies did not have statistical heterogeneity, so the fixed-effects model was selected for meta-analysis of the results. The combined effect size was [MD = −0.22, 95% CI (−1.00, 0.56)], that is, Tai Chi exercise has no significant improvement effect on SPPB and it cannot significantly improve the exercise capacity of stroke patients (Figure 12).

## 4. Discussion and Analysis

### 4.1. The Overall Analysis of the Impact of Tai Chi on the Balance Function and Exercise Ability of Stroke Patients

The results of this study prove that Tai Chi is able to significantly improve the balance function and exercise ability of stroke patients. This result is consistent with previous study results and again verifies the results of previous studies. Studies have shown that the stability of the core muscles of stroke patients is weak, and the stability of the trunk and pelvis is poor when completing antigravity activities, which leads to weakened exercise capacity [35] and decreased balance ability [36] during walking. As a low-intensity exercise method, Tai Chi can relax the tense muscles of patients, increase muscle flexibility and strength, improve the normal movement control ability of stroke patients, improve balance function, and inhibit the occurrence of abnormal postures and spasms, as well as have a positive effect on the life quality and psychological emotions of patients [7, 37, 38]. The study also found that regular Tai Chi exercises can promote the formation of functional nerve pathways, consolidate the efficiency of newly created or newly activated synapses, improve peripheral nerve conduction function, enhance the proprioception ability, promote exercise ability, and improve the quality of life [39].

### 4.2. The Effects of Physical Exercise Variables on the Balance Function and Exercise Ability among Stroke Patients

These studies show that Tai Chi exercises with different time durations all have a significant improvement on the balance function and exercise ability of stroke patients, among which >30 ≤ 60 min/time has the best effect on the patient's exercise ability. They also show that patients have not only improved their balance function and exercise performance but also significantly promoted the life quality index [40]. Another study found that, after Tai Chi exercises, upper limb function of stroke patients improved significantly, and the coordination and balance of the lower limbs also improved to some extent. Follow-up studies also found that their exercise ability and life activity ability also improve [41].

Tai Chi exercises at different frequencies each week have different effects on the balance function and exercise ability of stroke patients. Tai Chi frequency of twice a week by Hwang et al. [42] shows that the balance ability, coordination, and walking ability have significantly improved among stroke patients, which are essential for them to take care of themselves in daily life and prevent from falling down [43]. Studies have found that Tai Chi can increase the strength of the quadriceps and hamstrings, improve flexibility, thereby enhance the stability and balance of the knee joint, and improve the exercise ability of patients [44, 45]. In another frequency study by Wang et al. [46], after 5 times/wk of Tai Chi Cloud Hand training, the Berg Balance Scale score of the experimental group improved significantly, indicating that Tai Chi can promote the balance function of stroke patients. The study by Yang et al. [47] also found that 5 times/wk of Tai Chi exercises can adjust the exercise ability of the patients, thereby promoting gait stability.

The experimental cycles included in this study are all 12 weeks or less. The results show that the balance function and exercise ability of stroke patients in the treatment group improved significantly. A large number of previous studies have also disclosed the effect of a certain period of Tai Chi exercise on stroke patients. Hart et al. conducted 12-week Tai Chi exercise training for stroke patients on a community basis, whose results showed that the stroke patients improved in terms of balance function and exercise ability [48]. In another 12-week Tai Chi “Cloud Hand” intervention for stroke patients, the control group received routine rehabilitation training, evaluating the balance function and exercise ability through the Berg Balance Scale, as well as the standing and walking test, and the balance function and exercise ability of both groups improved. In the mean time, as the intervention period extended, Tai Chi had better influences on the balance function and exercise ability. Yu et al. analyzed the effect of Tai Chi for 24 weeks on the static balance of middle-aged and elderly people and found that the total length of the gravity center movement of the subjects, the peripheral area, and the shaking index of the surrounding area significantly improved, which shows the promotion of static balance ability of Tai Chi among the middle-aged and elderly people [49, 50].

## 5. Limitations

This study has certain limitations. First, the research methods of some included papers are not comprehensive, which did not explain the method of random allocation or blinding the subjects or evaluator. Therefore, the results may be inaccurate. Second, certain results of meta-analyses are highly heterogeneous. As a result, the research results may have a certain degree of bias. Third, some studies have small number of subjects and a small sample size, which suggests that more experimental studies are needed to prove our point of view.

## 6. Conclusions

Stroke has become a prominent problem that seriously endangers the health of middle-aged and elderly people, which features high morbidity, high disability, and high mortality. Tai Chi is considered as an effective nondrug intervention against hemiplegia, which is one of the most common sequelae of stroke patients. This study used the meta-analysis method to analyze the randomized controlled study on the intervention of Tai Chi on balance function and exercise ability among stroke patients, which further proved that Tai Chi can significantly enhance balance function and exercise ability of stroke patients. This study also analyzed the dose-response relationship of balance function and exercise ability when doing Tai Chi. Study results show that regular Tai Chi exercise once to twice a week, ≥5 times/wk, and >30 ≤ 60 min/time can improve the balance function and exercise capability of the stroke patients.

In the future, the study should consider the safety and effectiveness of Taijiquan intervention for stroke patients. Moreover, study in this filed should adopt comprehensive randomized controlled studies as much as possible, improve the methodological quality, strengthen the research on balance function and exercise ability of stroke patients, ensure the quality of evidence in research, enrich the research on different schemes of Tai Chi intervention, and improve the dose-response relationship between exercise variables of Tai Chi and balance function and Tai Chi and exercise ability among stroke patients.

## Figures and Tables

**Table 1:** Evidence level and recommendations grades of the Oxford Center for Evidence-Based Medicine.

Level of evidence	Contents	Recommended hierarchy
Ia	Systematic review with homogeneity of RCTs	I
Ib	Individual RCTs (with narrow confidence interval)	

IIa	Systematic reviews with homogeneity of cohort studies	II
IIb	Individual cohort study (including low-quality RCTs, e.g., <80% follow-up)	
IIc	Outcomes research	

IIIa	Systematic review of homogeneous case-control studies	III
IIIb	Individual case-control study	

III	Case series (and poor-quality cohort and case-control studies)	IV
—	Expert opinions without explicit critical appraisal	V

**Table 2:** Basic information of the included literature.

No.	Included literature	Nationality	Symptom	Number of subjects (T/C)	Age (T/C)	Percentage of men (T/C) (%)	Level of evidence
1	Au-Yeung et al. [16]	China	Stroke	59/55	61.7 ± 10.5	55.93	Ib
					65.9 ± 10.7	60.00	

2	Chan and Tsang [17]	China	Stroke	9/9	63.9 ± 6.1	55.56	Ib
					63.2 ± 6.0	44.44	

3	Huang et al. [18]	China	Stroke	14/14	62.21 ± 9.74	85.71	Ib
					59.93 ± 9.96	71.43	

4	Kim et al. [19]	Korea	Stroke	11/11	53.45 ± 11.54	63.60	Ib
					55.18 ± 10.20	54.50	

5	Taylor-Piliae and Coull [20]	The U.S.	Stroke	16/12	72.8 ± 10.1	62.5	Ib
					64.5 ± 10.9	58.33	

6	Taylor-Piliae et al. [21]	The U.S.	Stroke	53/48	71.5 ± 10.3	64.2	Ib
					68.2 ± 10.3	47.9	

7	Zheng et al. [22]	China	Stroke	85/85	61.01 ± 5.20	32.9	Ib
					60.73 ± 6.05	28.2	

8	Fan et al. [23]	China	Stroke	43/43	63.4 ± 5.0	67.44	Ib
					63.8 ± 5.3	69.77	

9	Fu and Zhang [24]	China	Stroke	30/30	59.7 ± 7.6	63.33	Ib
					60.3 ± 8.4	60	

10	Li et al. [25]	China	Stroke	30/30	71.03 ± 8.21	56.67	Ib
					71.06 ± 8.33	53.33	

11	Liu et al. [26]	China	Stroke	24/24	52.13 ± 14.13	58.33	Ib
					53.51 ± 12.63	45.83	

12	Xiao [27]	China	Stroke	46/46	57.51 ± 1.14	67.39	Ib
					57.68 ± 1.75	65.22	

13	Xu et al. [28]	China	Stroke	40/40	60.14 ± 10.25	55	Ib
					48.23 ± 12.32	40	

14	Yang and Liu [29]	China	Stroke	28/21	51.43 ± 47.63	60.71	Ib
					54.02 ± 38.41	61.90	

15	Yang and Tang [30]	China	Stroke	28/21	51.43 ± 15.63	60.71	Ib
					54.85 ± 11.85	66.67	

16	Yang et al. [31]	China	Stroke	30/30	58 ± 11.27	66.67	Ib
					60.07 ± 7.87	60	

17	Zhang et al. [32]	China	Stroke	20/20	67.80 ± 12.22	50	Ib
					66.65 ± 10.49	55	

18	Zhao et al. [33]	China	Stroke	30/30	53.85 ± 11.69	66.67	Ib
					51.38 ± 14.83	63.33	

19	Yi et al. [34]	China	Stroke	66/66	48.78 ± 13.52	63.64	Ib
					47.69 ± 14.91	59.09	

T: treatment group; C: control group.

**Table 3:** Sports intervention features in the included literature.

No.	Included in the study	Intervention measures		Intervention elements			Outcome indicators
		Treatment group	Control group	Control group	Frequency (times/wk)	Time (min/time)	
1	Au-Yeung et al. [16]	Tai Chi	Regular activity	12	4	60	TUG
2	Chan and Tsang [17]	Tai Chi	Regular activity	12	2	60	Gravity swing test
3	Huang et al. [18]	Tai Chi	Regular activity	12	3	40	FMA
4	Kim et al. [19]	Tai Chi + physiotherapy	Physiotherapy	6	2	60	TUG, 10 mwt
5	Taylor-Piliae and Coull [20]	Tai Chi	Health education	12	3	60	SPPB
6	Taylor-Piliae et al. [21]	Tai Chi	Regular activity	12	3	60	SPPB
7	Zheng et al. [22]	Tai Chi	Regular activity	12	5	60	6 mwt
8	Fan et al. [23]	Tai Chi + conventional rehabilitation	Conventional rehabilitation	12	3	90	BBS, TUG, and FMA
9	Fu and Zhang [24]	Tai Chi + conventional rehabilitation	Conventional rehabilitation	8	6	40	BBS
10	Li et al. [25]	Tai Chi + medicine	Medicine	12	2	60	FMA
11	Liu et al. [26]	Tai Chi + conventional rehabilitation	Conventional rehabilitation	12	7	30	BBS
12	Xiao [27]	Tai Chi + conventional rehabilitation	Conventional rehabilitation	—	5	40	FMA
13	Xu et al. [28]	Tai Chi + physiotherapy	Physiotherapy	12	7	40	BBS
14	Yang and Liu [29]	Tai Chi + conventional rehabilitation	Conventional rehabilitation	8	3	40	FMA
15	Yang and Tang [30]	Tai Chi + comprehensive rehabilitation	Comprehensive rehabilitation	8	5	40	FMA and BBS
16	Yang et al. [31]	Tai Chi + traditional walking	Traditional walking	—	7	15 (Tai Chi) + 45 (traditional walking)	FMA
17	Zhang et al. [32]	Tai Chi + conventional rehabilitation	Conventional rehabilitation	3	7	30	FMA
18	Zhao et al. [33]	Tai Chi + conventional rehabilitation	Conventional rehabilitation	8	5	30	FMA
19	Yi et al. [34]	Tai Chi + conventional rehabilitation	Conventional rehabilitation	12	2	60	BBS, FMA, and swing test

“—” indicates that there is no description in the literature; TUG: timed up and go test; FMA: Fugl-Meyer motor function score scale; 10 mwt: 10 m walking test; SPPB: short physical performance battery; 6 mwt: 6 m walking test; BBS: Berg Balance Scale.
